# Factors Associated with Treatment Outcome in Patients with Nontuberculous Mycobacterial Pulmonary Disease: A Large Population-Based Retrospective Cohort Study in Shanghai

**DOI:** 10.3390/tropicalmed7020027

**Published:** 2022-02-15

**Authors:** Li-Ping Cheng, Shan-Hao Chen, Hai Lou, Xu-Wei Gui, Xiao-Na Shen, Jie Cao, Wei Sha, Qin Sun

**Affiliations:** Clinical and Research Center for Tuberculosis, Shanghai Key Laboratory of Tuberculosis, Shanghai Pulmonary Hospital, School of Medicine, Tongji University, Shanghai 200433, China; clptina99@163.com (L.-P.C.); cshddd@163.com (S.-H.C.); louhai0201@163.com (H.L.); 13611914313@139.com (X.-W.G.); xiaonashen1003@163.com (X.-N.S.); roundyuan511@163.com (J.C.)

**Keywords:** nontuberculous mycobacteria, lung disease, risk factors, treatment outcome

## Abstract

Infectious diseases caused by nontuberculous mycobacteria (NTM) are increasingly common. This retrospective cohort study examined factors associated with outcomes in patients from Shanghai who had NTM pulmonary disease (NTMPD) from January 2014 to December 2018. The causative bacterial species, drug susceptibility test results, treatment outcomes, sputum culture conversion rate, and risk factors associated with treatment failure were determined. The most common species were *Mycobacterium avium* complex (MAC) (50%), *M. abscessus* (28%), and *M. kansasii* (15%). Over five years, the proportions of *M. kansasii* and *M. abscessus* increased, and that of MAC decreased. The treatment success rate was significantly greater for patients infected with *M. kansasii* (89.9%) than MAC (65.0%, *p* < 0.001) and *M. abscessus* (36.1%, *p* < 0.001). Multivariate analysis indicated the risk factors for treatment failure were pathogenic NTM species (*M. abscessus*: aOR = 9.355, *p* < 0.001; MAC: aOR = 2.970, *p* < 0.001), elevated ESR (>60 mm/h: aOR = 2.658, *p* < 0.001), receipt of retreatment (aOR = 2.074, *p* < 0.001), and being middle-aged or elderly (>60 years-old: aOR = 1.739, *p* = 0.021; 45–60 years-old: aOR = 1.661, *p* = 0.034). The main bacterial species responsible for NTMPD were MAC, *M. abscessus*, and *M. kansasii*. Patients who were infected by *M. abscessus* or MAC, with elevated ESR, received retreatment, and were middle-aged or elderly had an increased risk of treatment failure.

## 1. Introduction

Infection by nontuberculous mycobacteria (NTM) has become a major public health problem in many geographical regions [1,2,3]. The detection of NTM has increased due to the increased prevalence of diseases that cause immunodeficiency, such as HIV/AIDS; the increased use of immunosuppressive agents or hormones; and improvements in bacterial identification, such as genetic sequencing. Data from previous epidemiological surveys of tuberculosis (TB) in China showed that the rate of NTM isolation was 4.3% in 1979, 11.1% in 2000, and 22.9% in 2010. There are more than 200 known NTM species worldwide, most of which are parasitic bacteria, although only a few are pathogenic to humans. The common pathogenic NTM species are *Mycobacterium avium* complex (MAC), *M. abscessus*, *M. kansasii*, and *M. xenopi* [4]. The different NTM species vary in their geographical distributions [5,6]. For example, MAC, *M. abscessus*, *M. fortuitum*, and *M. kansasii* are commonly reported in the Pacific region [7]. China has a heavy burden of NTM diseases. However, current epidemiological data on NTM pulmonary disease (NTMPD) in China, such as the distribution and incidence of different causative species, are very limited.

Most NTM are naturally resistant to commonly used anti-mycobacterial drugs [4], and treatment with multiple drugs is often necessary. Typically, the treatment course for NTM is very long and the cost is high [8]. Moreover, the side effects of antibiotics are often substantial [9], and the development of new drugs has been slow. As a result, the clinical efficacies of available treatments are unsatisfactory and the recurrence rate is very high. Screening patients for risk factors associated with treatment failure is important because it may help predict disease prognosis, prevent disease progression, improve therapeutic outcome, and improve the allocation of resources. Very few previous studies examined the risk factors for treatment failure in patients with NTMPD.

We conducted a large retrospective cohort study from 2014 to 2018 to examine treatment outcomes in patients from Shanghai who had NTMPD. We systematically investigated the incidence, causative bacterial species, drug susceptibility test results, treatment outcomes, sputum culture conversion rate, and risk factors associated with treatment failure. Our findings may help improve the clinical management of patients with NTMPD.

## 2. Materials and Methods

### 2.1. Patient Selection

**Figure 1:**
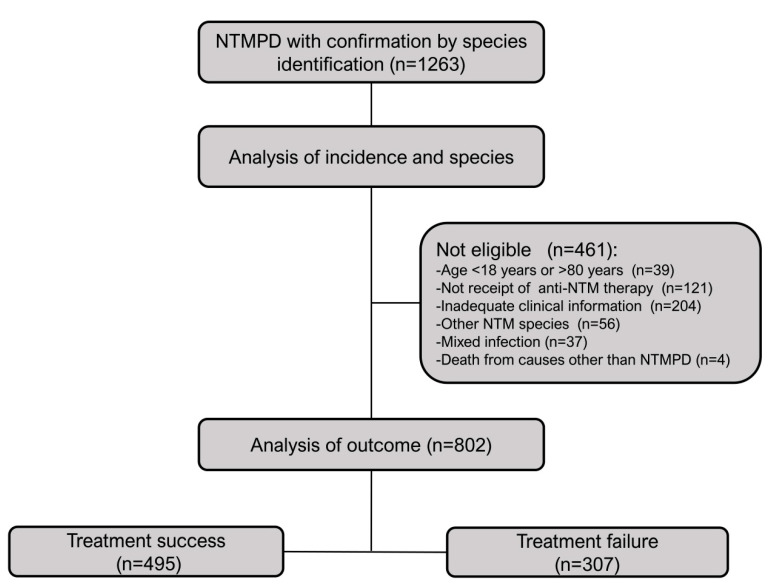
Identification and disposition of patients with NTMPD. NTMPD: nontuberculous mycobacterial pulmonary disease, and NTM: nontuberculous mycobacteria.

Data of 1263 NTMPD patients who were treated at Shanghai Pulmonary Hospital between January 2014 and December 2018 were retrospectively collected. This study was approved by the Ethics Committees of Shanghai Pulmonary Hospital. Figure 1 shows the disposition of the initial 1263 patients and the criteria used to enroll 802 patients. All included patients were: (i) 18 to 80 years old and met the diagnostic criteria of the ATS/ERS/ESCMID/IDSA clinical practice guideline [4] and the 2020 Guideline for the Diagnosis and Treatment of Non-tuberculous Mycobacteria Diseases from the Chinese Medical Association; (ii) provided sputum or bronchoalveolar lavage fluid (BALF) specimens that were subjected to polymerase chain reaction (PCR) reverse dot blot hybridization for bacterial species identification, with confirmation of results; and (iii) received regular treatment under the guidance of physicians and had complete clinical data available. Patients were excluded if they were pregnant or lactating women; had an HIV infection; or had co-infection with two *Mycobacterium* species.

### 2.2. Culture Methods, Species Identification, and Drug Susceptibility Testing

Sputum or BALF specimens from patients were cultured and identified as NTM at least twice using the BACTEC MGIT 960 method in accordance with the Chinese Tuberculosis Bacteriological Examination Procedure. Drug susceptibility tests for isoniazid, rifampicin, ethambutol, amikacin, and ofloxacin were performed. Molecular biological detection of NTM species in sputum or BALF specimens was performed using PCR-reverse dot blot hybridization in accordance with the instructions of a Mycobacterium identification gene detection kit (Yaneng BIOscience Co., Shenzhen, China).

### 2.3. Study Design and Follow-Up

This was a retrospective cohort study. For each patient, the onset of anti-NTM treatment was used as the start-point, and death, loss to follow-up, or end of follow-up (December 2020) as the end-point. All enrolled patients were inpatients and were followed up through the outpatient department of the hospital after discharge. All data were recorded in detail, including demographic data, clinical manifestations, laboratory test results, imaging findings, and treatment outcomes. All enrolled patients received anti-NTM therapy. The selection of anti-NTM drugs was based on the results of drug susceptibility tests and 2012 Expert Consensus of Chinese Medical Association. Briefly, patients with *M. kansasii* infections were treated with a regimen containing at least three of the following drugs: isoniazid (5 mg/kg), rifampicin (10 mg/kg), ethambutol (15 mg/kg), moxifloxacin (400 mg/day), clarithromycin (1000 mg/day) or azithromycin (500 mg/day). Patients with MAC infections were treated with at least three of the following drugs: rifampicin or rifabutin (300 mg/day), ethambutol, clarithromycin or azithromycin, moxifloxacin, and amikacin (400 mg/day). Patients with *M. abscessus* infections were treated with at least four of the following drugs: clarithromycin or azithromycin, amikacin, cefoxitin (200 mg/kg), faropenem (300 mg/day), linezolid (600 mg/day), and clofazimine (150 mg/day). The patients were initiated with anti-NTM therapy as soon as they met the diagnostic criteria of NTMPD according to the guideline of ATS/ERS/ESCMID/IDSA and Chinese Medical Association and a careful clinical assessment. The duration of treatment for patients with rifampicin-sensitive M. kansasii infection was at least one year, and the duration was at least one year after sputum culture conversion in patients with rifampicin-resistant *M. kansasii*, MAC, and *M. abscessus* pulmonary disease.

The results of sputum smears, NTM cultures, drug susceptibility tests, and chest computed tomography (CT) within 1 month before the onset of anti-NTM treatment were used as baseline. Routine blood indexes, liver and renal function tests, and electrolytes were assessed once per month after treatment onset. Sputum smears, NTM cultures, drug susceptibility tests, and chest CT examinations were performed every 3 months after treatment onset. Chest CT examinations were also performed every 6 months during the first year after treatment cessation, and then every year until the end of the study. Sputum examinations were performed if lesions increased, if new pulmonary cavities appeared, or if pulmonary cavities worsened.

To monitor adverse drug reactions during the anti-NTM therapy, patients received routine monthly examinations (e.g., blood work, and liver and renal function tests), and were encouraged to report any new symptoms (e.g., nausea, vomiting, and lack of appetite) that might be related to drug therapy. The diagnosis and management of adverse reactions related to the use of anti-NTM drugs were according to the Expert Consensus of the Chinese Medical Association. These adverse reactions were mainly: (i) drug induced liver injury, defined by serum alanine aminotransferase (ALT) level more than five times the upper limit of normal (ULN), or alkaline phosphatase (ALP) level more than two times the ULN, or serum ALT level more than three times the ULN combined with total bilirubin (TBIL) level more than two times the ULN, after exclusion of other causes; (ii) cytopenia, defined by anemia (hemoglobin <11 g/dL), neutropenia (<1500 neutrophils/µL), or thrombocytopenia (<100,000 platelets/µL); (iii) skin allergic rash, defined by skin pruritus and rash after taking an anti-NTM drug that was significantly relieved after drug discontinuation; and (iv) kidney injury, defined by creatinine clearance below 90 mL/min during anti-NTM therapy, after exclusion of other causes.

### 2.4. Treatment Outcome and Prognosis

Recurrence within 1 year was defined as the re-emergence of at least two positive results for the causative species from cultures of respiratory samples within 1 year after cessation of antimycobacterial treatment.

Culture conversion was defined as at least three consecutive negative mycobacterial cultures from respiratory samples, collected at least 4 weeks apart, during antimycobacterial treatment. The sampling date of the first negative culture was the date of culture conversion.

The treatment outcome categories were based on the 2020 Guideline of Chinese Medical Association, which is basically the same as the NTM-NET consensus [10] in principle. The outcomes in this study were classified as: (i) microbiological cure (multiple consecutive negative and no positive cultures with the causative species from respiratory samples after culture conversion and until the end of antimycobacterial treatment); (ii) cure (fulfillment of criteria for microbiological cure and clinical condition improvement); (iii) failure (no culture conversion, or the re-emergence of positive cultures with the causative species from respiratory samples after culture conversion while the patient is still on treatment); and (iv) death due to NTMPD. For statistical analyses, the “treatment success” group included “microbiological cure” and “cure” and the “treatment failure” group included “failure” and “death”.

### 2.5. Statistical Analysis

SPSS version 20.0 (SPSS Inc., Chicago, IL, USA) was used for statistical analyses. The χ^2^ test was used to compare the treatment outcomes and prognoses of patients with the three most common NTM species, and a *p* value below 0.05 in a two-sided *t*-test indicated statistical significance. The Bonferroni method was used for pairwise comparisons. The changes over time in the sputum culture conversion rates of patients infected with *M. kansasii*, MAC, or *M. abscessus* were plotted using the Kaplan–Meier method, and the statistical significance of differences was determined using the log-rank test. Unconditional binary logistic regression was used for univariate and multivariate analyses to identify risk factors for treatment failure. The unadjusted odds ratio (OR) and 95% confidence interval (CI) for each variable were first calculated using univariate regression. Multivariate regression was then performed using the forward conditional method. The test level (α) for including a variable was 0.05, and the test level (α) for excluding a variable was 0.10. The adjusted OR (aOR) and 95% CI was calculated for each included variable.

## 3. Results

### 3.1. Abundances of Different NTM Species in Patients with NTMPD

**Figure 2:**
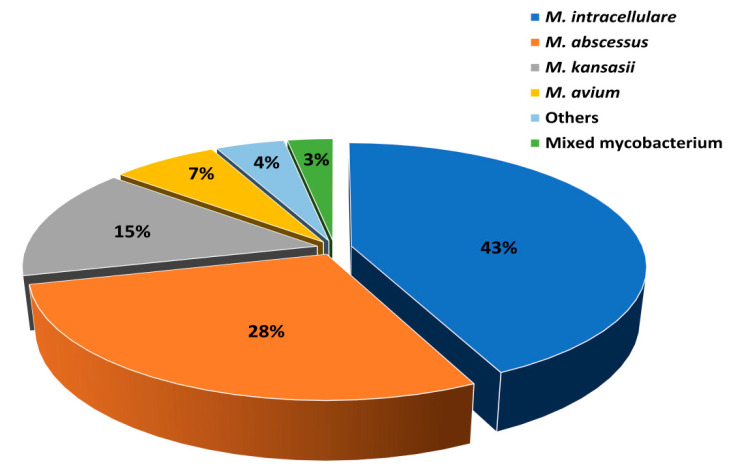
Abundances of different NTM species in patients diagnosed with NTMPD. NTM: nontuberculous Mycobacteria, and NTMPD: nontuberculous mycobacterial pulmonary disease.

From January 2014 to December 2018, 1263 patients at the Shanghai Pulmonary Hospital had NTMPD, with confirmation by species identification (Figure 2). We identified the causative species as *M. intracellulare* (542, 43%), *M. abscessus* (357, 28%), *M. kansasii* (185, 15%), *M. avium* (86, 7%), other NTM species (56, 4%), and mixed infections (37, 3%). The 56 other species were *M. szulgai* (8), *M. scrofulaceum* (11), *M. chelonae* (12), *M. xenopi* (8), *M. gordonae* (4), *M. fortuitum* (5), unidentified NTM species (6), and *M. malmoense* (2). The 37 cases of mixed infections were *M. tuberculosis* with NTM (8), *M. abscessus* with *M. intracellulare* (22), *M. abscessus* with *M. avium* (4), and *M. scrofulaceum* with *M. intracellulare* (3).

**Figure 3:**
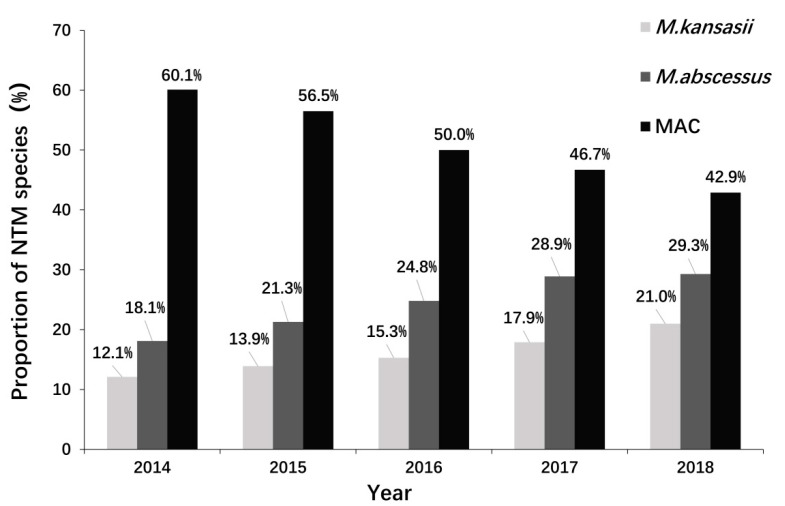
Changes in the abundances of major NTM species in patients with NTMPD from 2014 to 2018. NTM: nontuberculous mycobacteria, MAC: Mycobacterium avium complex.

Among all positive mycobacterial cultures from samples of sputum and BALF, the overall NTM positivity rate increased steadily over time (2014: 9.1%; 2015: 10.9%; 2016: 12.1%; 2017: 13.7%; 2018: 15.5%). We further analyzed the three most common NTM species (MAC, *M. abscessus*, *M. kansasii*). From 2014 to 2018, there were steadily increasing percentages of patients with *M. kansasii* and *M. abscessus* infections, and a decreasing percentage of patients with MAC infections (Figure 3).

### 3.2. Drug Susceptibility of the Three Major NTM Species

We analyzed the drug resistance of the NTM isolates from the 802 included patients (Table 1). *M. kansasii* generally had the lowest resistance to the five different drugs (amikacin: 2.5%, isoniazid: 25.3%, rifampicin: 13.9%, ethambutol 19.0%, and ofloxacin: 12.7%), MAC generally had intermediate resistance (amikacin: 21.6%, isoniazid: 85.4%, rifampicin: 39.6%, ethambutol 27.8%, and ofloxacin: 54.0%), and *M. abscessus* generally had high resistance (amikacin: 25.1%, isoniazid: 100%, rifampicin: 99.1%, ethambutol 98.2%, and ofloxacin: 99.1%).

### 3.3. Outcomes and Sputum Culture Conversion Rates of Isolates from NTMPD Patients Infected with the Three Major Species

The overall median time of treatment was 19.1 months (95%CI: 18.6, 19.6) in the treatment success group and 23.9 months (95%CI: 23.8, 24.1) in the treatment failure group (Table 2). In treatment success and failure groups, we also compared the median duration of therapy for patients with *M. kansasii* infections (13.5 months [95%CI: 12.9, 14.0] vs. 22.3 months [95%CI: 21.0, 23.6]), MAC infections (18.6 months [95%CI: 16.5, 19.6] vs. 23.9 months [95%CI: 23.7, 24.1]), and *M. abscessus* infections (21.4 months [95%CI: 20.9, 22.0] vs. 24.2 months [95%CI: 24.0, 24.4]).

Among the 802 patients with NTMPD, we classified 495 patients (61.7%) as “treatment success” and 307 patients (38.3%) as “treatment failure”. There were 21 cases (2.6%) of recurrence within 1 year, one involving *M. kansasii* (0.6%), nine recurrences involving MAC (2.1%), and eleven recurrences involving *M. abscessus* (4.8%).

**Figure 4:**
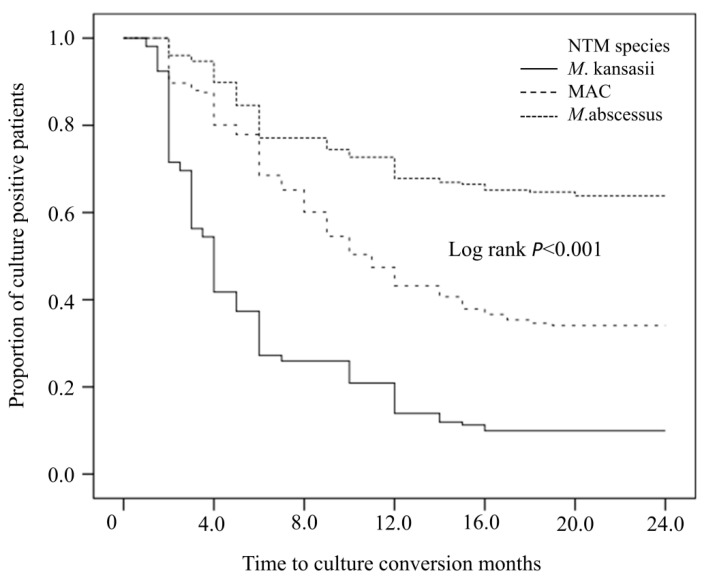
Sputum culture conversion rates of patients infected by *M. Kansasii*, MAC, and *M. abscessus*. NTM: nontuberculous mycobacteria, and MAC: *Mycobacterium avium* complex.

We also compared the treatment outcomes and sputum culture conversion rates of patients infected with the three major NTM species. The treatment success rate was 89.9% for patients with *M. kansasii*, 65.0% for patients with MAC, and 36.1% for patients with *M. abscessus*. Pairwise comparisons indicated the success rate was significantly higher for patients with *M. kansasii* than for patients with the other species (both *p* < 0.001, Table 2). However, the three groups had no statistically significant differences in recurrence within one year. The median time to sputum culture conversion was 4 months (95%CI: 3.472, 4.528) for *M. kansasii* infections, 10 months (95%CI: 7.107–12.893) for MAC infections, and 24 months (no conversion after treatment) for *M. abscessus* infections. The sputum negative conversion rate was significantly higher for *M. kansasii* (89.9%, Log rank test: *p* < 0.001) than for MAC (65.0%) and *M. abscessus* (36.1%; Figure 4).

### 3.4. Univariate Analysis of Factors Associated with Treatment Failure in NTMPD Patients

We performed a univariate analysis to identify factors at baseline that were significantly associated with treatment failure (Table 3). Treatment failure was more common in patients who were middle-aged and elderly (45–60 years-old and >60 years-old), were female, received retreatment, had the complication of chronic obstructive pulmonary disease (COPD), had bronchiectasis, had pulmonary cavities, had involvement of multiple lung fields (3–4 lung fields or 5–6 lung fields), had elevated ESR (> 60 mm/h), were infected by different NTM species (MAC, *M. abscessus*), and had gastrointestinal reactions (all *p* < 0.05).

### 3.5. Multivariate Analysis of Factors Associated with Treatment Failure in NTMPD Patients

A multivariate binary logistic regression analysis indicated that four risk factors were significantly and independently associated with treatment failure (Table 4). In order of risk, these factors were: pathogenic NTM species (*M. abscessus*: aOR = 9.355, *p* < 0.001; MAC: aOR = 2.970, *p* < 0.001), elevated ESR (>60 mm/h: aOR = 2.658, *p* < 0.001), receipt of retreatment (aOR = 2.074, *p* < 0.001), and being middle-aged or elderly (45–60 years-old: aOR = 1.661, *p* = 0.034; >60 years-old: aOR = 1.739, *p* = 0.021).

## 4. Discussion

The present investigation was a large retrospective cohort study of 1263 patients diagnosed with NTMPD in Shanghai from 2014 to 2018 that determined the overall incidence, bacterial species distribution, treatment outcome, and factors associated with treatment outcome. The major causative species were MAC, *M. abscessus*, and *M. kansasii*. The treatment success rate and sputum culture conversion rate were much higher in patients with *M. kansasii* infections than in those with infections by the other two species. Among all patients, infection by *M. abscessus*, infection by MAC, having an elevated ESR, receipt of retreatment, and being middle-aged or elderly had significant and positive associations with treatment failure. These findings provide important additional information regarding the clinical prognosis in patients with NTMPD, and may help to improve treatment outcomes in these patients.

The most common NTM species in the world are MAC, *M. abscessus*, and *M. kansasii* [11], identical to our findings in NTMPD patients in Shanghai from 2014 to 2018. However, we identified changing percentages of these species over time. In particular, the percentages of *M. kansasii* and *M. abscessus* increased, and the percentage of MAC decreased. We also found that infection by multiple NTM species was common, similar to a 2020 study that was conducted in Chongqing (China) [12]. However, the distributions of the different NTM species vary in different countries. In Europe and North America, MAC, *M. gordonae*, *M. xenopi*, and *M. fortuitum* are the most common, but in South America, MAC, *M. kansasii*, *M. gordonae*, and *M. fortuitum* are the most common [13]. These geographic differences may be due to various factors, such as differences in temperature, humidity, and the living habits of local residents.

Previous studies reported that many NTM species had natural drug resistance, especially to conventional first-line anti-TB drugs [14,15]. Our results are consistent with these findings, and we also found that drug resistance was particularly notable in *M. abscessus*. This drug resistance may be due to the cell wall acting as a natural barrier, drug efflux systems, drug inactivation, mutation or deletion of drug target sites, plasmids that confer resistance [16], or a combination of factors. Therefore, in clinical practice, NTMPD patients require long treatment courses, although treatment efficacy is often low and the recurrence rate is often high. The present study showed that there were significant differences in the treatment success rates and sputum culture conversion rates of patients infected with different NTM species. In particular, patients infected with *M. kansasii* had significantly better outcomes than patients infected with MAC or *M. abscessus*.

Our large retrospective cohort study also analyzed the risk factors for treatment failure. We found that the risk of treatment failure was greater for patients infected with *M. abscessus* (aOR = 9.355) and MAC (aOR = 2.970) compared to those infected with *M. kansasii*, consistent with previous studies [17,18]. Among our three most common NTM species, *M. kansasii* was the most sensitive to common anti-TB drugs. Macrolides, quinolones, aminoglycosides, and sulfonamides are also effective against *M. kansasii*, and it is easier to develop a regimen that includes more than three effective drugs. Relative to *M. kansasii*, MAC has much greater rates of resistance to amikacin, isoniazid, rifampicin, ethambutol, doxycycline, clarithromycin, linezolid, and moxifloxacin [19]. *M. abscessus* is the most problematic species because of its high rates of resistance to rifamycin, macrolides (including clarithromycin and azithromycin), and other key therapeutic drugs [20,21], potentially due to induced drug resistance or drug resistance caused by mutations [22]. Our results showed that *M. abscessus* had extremely high resistance to all first-line anti-TB drugs, and was only sensitive to amikacin (74.9%). The 2020 American Thoracic Society Guidelines recommended linezolid and clofazimine for treatment of *M. abscessus* infections [4]. In the present study, only 21 patients (9.3%) with *M. abscessus* infections received linezolid and only 19 patients (8.4%) received clofazimine because these two drugs were not widely used for NTMPD treatment in Shanghai during the study period. We believe this might be part of the reason for our low treatment success rate in patients who had *M. abscessus* infections.

Previous studies of MAC lung disease (MAC-LD) found that females had worse prognoses [23,24]. Studies in the United States and Denmark showed that the mortality rate was significantly higher for elderly women with NTMPD [25,26]. Our multivariate results indicated that treatment failure correlated with age, but not with female sex. Relative to patients younger than 45 years, the aOR for treatment failure was 1.661 for patients aged 45 to 60 years and 1.739 for patients older than 60 years.

A recent study suggested that a history of previous NTMPD might be related to unfavorable treatment outcome in patients with lung disease due to *M. abscessus* infection [27]. In agreement, we found that the aOR for treatment failure was 2.074 for those who received previous NTMPD treatment. This may be because the initial structural damage of lung tissue caused by the sequelae of NTMPD, which could reduce local drug bioavailability. In addition, during NTMPD retreatment, increased drug resistance, high treatment cost, long treatment course, and psychological factors of the patient may contribute to poor patient compliance and increased treatment difficulties.

Gochi et al. [28] found that patients who were older and had lower BMI values were more likely to experience aggravation of MAC-LD and increased levels of serum inflammatory indicators. We found no effect of BMI on outcome, but patients with an elevated ESR had a notable risk for poor outcome (>60 mm/h: aOR = 2.658). ESR and C-reactive protein are common inflammatory markers, and increased levels may reflect a systemic inflammatory response caused by NTMPD infection, and often indicate disease progression. Previous research indicated that an ESR above 50 mm/h was a negative prognostic indicator of radiologic deterioration in NTMPD complicated with rheumatoid arthritis [29]. A previous animal study of MAC-LD found that the level of certain proinflammatory and anti-inflammatory cytokines, such as TNF-α and gamma interferon, were greater in mice with severe or advanced infections [30].

An increasing number of studies have confirmed that certain chest imaging features, such as bronchiectasis and pulmonary cavitations, are related to the prognosis of patients with NTMPD [24,31,32]. Similarly, patients with NTMPD often present with bronchiectasis [33,34,35]. There is also evidence that the occurrence of NTM may correlate with alpha-1-antitrypsin deficiency in patients with bronchiectasis [36] and that bronchiectasis is a predisposing factor for reinfection by NTM species [37]. Our univariate analysis indicated bronchiectasis was a significant risk factor for treatment failure, but it was not significant in the multivariate analysis. Our results also showed that infection by *M. abscessus* had a significant correlation with treatment failure. A high proportion of patients infected by *M. abscessus* have bronchiectasis, therefore, we consider bronchiectasis as a confounding factor for treatment failure.

The current study has some limitations. It had a retrospective cohort design, and complete clinical data, especially the susceptibility to certain drugs (clarithromycin, azithromycin doxycycline, clarithromycin, and linezolid) were not available. The subspecies of *M. abscessus* were not identified because of technological limitations. Therefore, we suggest that the results of this study need verification by large, multi-center, prospective cohort studies.

## 5. Conclusions

This study found that the major species causing NTMPD in Shanghai from 2014 to 2018 were MAC, *M. abscessus*, and *M. kansasii*. The treatment success rate in patients with *M. kansasii* infections was much higher than in those infected by the other two species. Infection by *M. abscessus* or MAC, elevated ESR, receipt of retreatment, and being middle-aged or elderly were independent risk factors for treatment failure. We, therefore, recommend that when clinicians encounter a patient with any of these conditions, they should carefully evaluate disease status and perform drug susceptibility tests, and then implement the most appropriate treatment regimen. The Directly Observed Treatment Strategy, which is applied in TB management, should be considered for patients with NTMPD, and these patients should be closely monitored for adverse drug reactions and compliance to minimize the risk of treatment failure. Our results provide valuable insights for predicting the prognosis and improving treatment outcome in patients with NTMPD.

## Figures and Tables

**Table 1:** Drug resistance rates of the three major NTM species.

Drug	*M. kansasii* (n = 158)	MAC (n = 417)	*M. abscessus* (n = 227)
	n (%)	n (%)	n (%)
Am	4 (2.5)	90 (21.6)	57 (25.1)
INH	40 (25.3)	356 (85.4)	227 (100)
RFP	22 (13.9)	165 (39.6)	225 (99.1)
EMB	30 (19.0)	106 (27.8)	223 (98.2)
Ofx	20 (12.7)	225 (54.0)	225 (99.1)

Am: amikacin, INH: isoniazid, RFP: rifampicin, EMB: ethambutol, and Ofx: ofloxacin. MAC: Mycobacterium avium complex.

**Table 2:** Outcomes of NTMPD patients infected with the three major species.

Outcome	*M. Kansasii* (n = 158)	MAC (n = 417)	*M. abscessus* (n = 227)	χ^2^	*p* ^a^
	n (%)	n (%)	n (%)		
Success	142(89.9)	271(65.0)	82(36.1)	117.841	<0.001
Failure	16(10.1)	146(35.0)	145(63.9)	117.841	<0.001
Recurrence (within 1 year)	1(0.6)	9(2.1)	11(4.8)	--	0.027 ^a^

^a^ *p*-value corrected using Fisher’s exact method when the expectation was below 5, --: no statistical value. MAC: *Mycobacterium avium* complex.

**Table 3:** Univariate analysis of factors associated with treatment failure in patients with NTMPD.

Factor	Success (n = 495)	Failure (n = 307)	OR	95% CI	*p* Value
	n (%)	n (%)			
Age, years					
18~44	145(29.3)	46(15.0)	1(ref)		
45~60	161(32.5)	104(33.9)	2.036	1.347, 3.078	0.001
>60	189(38.2)	157(51.1)	2.618	1.767, 3.881	<0.001
Female	244(49.3)	186(60.6)	1.581	1.185, 2.111	0.002
Retreatment NTMPD	131(26.5)	155(50.5)	2.881	2.121, 3.912	<0.001
Smoking history	50(10.1)	22(7.2)	0.571	0.369, 1.732	0.800
Rural residence	215(43.4)	141(45.9)	1.112	0.831, 1.488	0.476
History of pulmonary TB	137(27.7)	94(30.6)	1.145	0.836, 1.568	0.399
COPD	27(5.4)	32(10.4)	2.167	1.279, 3.670	0.004
Pneumoconiosis	17(3.4)	10(3.3)	1.193	0.728, 2.095	0.893
Long-term use of corticosteroid/immunosuppressant	13(2.6)	12(3.9)	1.508	0.679, 3.349	0.313
Diabetes Mellitus	34(6.9)	16(5.2)	0.746	0.404, 1.375	0.347
BMI (kg/m^2^)					
≥18.5	322(65.1)	180(58.6)	1(ref)		
<18.5	173(34.9)	127(41.4)	1.313	0.980, 1.760	0.068
Pulmonary cavities	249(50.3)	180(58.6)	1.404	1.056, 1.873	0.020
Bronchiectasis	306(61.8)	218(71.0)	1.513	1.114, 2.055	0.008
Involvement of lung fields					
1~2	146(29.5)	51(16.6)	1(ref)		
3~4	115(23.2)	73(23.8)	1.817	1.178, 2.802	0.007
5~6	234(47.3)	183(59.6)	2.239	1.542, 3.251	<0.001
ESR(mm/L)					
<15	168(33.9)	73(23.8)	1(ref)		
15~60	272(54.9)	164(53.4)	1.388	0.991, 1.942	0.056
>60	55(11.2)	70(22.8)	2.929	1.872, 4.582	<0.001
Anemia	417(84.2)	256(83.4)	0.927	0.630, 1.364	0.700
Albumin (g/L)					
>34	430(86.9)	247(80.5)	1(ref)		
25~34	53(10.7)	51(16.6)	1.304	0.784, 2.171	0.306
<25	12(2.4)	9(2.9)	2.036	1.169, 3.522	0.813
Flow Cytometry					
CD4+ (<0.34)	307(62.0)	172(56.0)	1.276	0.845, 1.926	0.247
CD8+ (<0.25)	147(29.7)	94(30.6)	0.959	0.617, 1.490	0.853
CD4+/CD8+ (<0.68)	482(97.4)	296(96.4)	1.437	0.431, 4.793	0.555
Bacterial species					
*M. kansasii*	142(28.7)	16(5.2)	1(ref)		
MAC	271(54.7)	146(47.6)	4.781	2.746, 8.326	<0.001
*M. abscessus*	82(16.6)	145(47.2)	15.694	8.756, 28.128	<0.001
Treatment regimen changes	143(28.9)	108(21.8)	1.336	0.985, 1.811	0.062
Adverse drug reactions					
Hepatotoxicity	43(8.7)	24(4.8)	0.891	0.529, 1.501	0.666
Cytopenia	43(8.7)	21(6.8)	0.618	0.255, 1.498	0.287
Hypersensitivity	17(3.4)	14(4.6)	1.344	0.653, 2.766	0.423
Gastrointestinal	21(4.2)	26(8.5)	2.088	1.153, 3.781	0.015

95% CI: 95% confidence interval, OR: unadjusted odds ratio, NTMPD: nontuberculous mycobacterial pulmonary disease, TB: tuberculous, COPD: chronic obstructive pulmonary disease, BMI: body mass index, ESR: erythrocyte sedimentation rate, and MAC: *Mycobacterium avium* complex.

**Table 4:** Multivariate analysis of factors associated with treatment failure in patients with NTMPD.

Factor	aOR	95% CI	*p* Value
Retreatment NTMPD	2.074	1.470, 2.926	<0.001
Age, years			
18~44	1(ref)		
45~60	1.661	1.038, 2.659	0.034
>60	1.739	1.088, 2.778	0.021
ESR(mm/h)			
<15	1(ref)		
15~60	1.185	0.800,1.755	0.398
>60	2.658	1.560, 4.529	<0.001
Bacterial species			
*M. kansasii*	1(ref)		
MAC	2.970	1.620, 5.443	<0.001
*M. abscessus*	9.355	4.977, 17.584	<0.001

95% CI: 95% confidence interval, aOR: adjusted odds ratio, NTMPD: nontuberculous mycobacterial pulmonary disease, ESR: erythrocyte sedimentation rate, and MAC: *Mycobacterium avium* complex.

## Data Availability

The datasets used and/or analyzed during the current study are available from the corresponding author on reasonable request. (Q.S., Email: sunqinbonjour@163.com).

## References

[1] Haworth C.S., Banks J., Capstick T., Fisher A.J., Gorsuch T., Laurenson I.F., Leitch A., Loebinger M.R., Milburn H.J., Nightingale M. (2017). British Thoracic Society guidelines for the management of non-tuberculous mycobacterial pulmonary disease (NTM-PD). Thorax.

[2] Ratnatunga C.N., Lutzky V.P., Kupz A., Doolan D.L., Reid D.W., Field M., Bell S.C., Thomson R.M., Miles J.J. (2020). The Rise of Non-Tuberculosis Mycobacterial Lung Disease. Front. Immunol..

[3] Mirsaeidi M., Farshidpour M., Allen M.B., Ebrahimi G., Falkinham J.O. (2014). Highlight on Advances in Nontuberculous Mycobacterial Disease in North America. BioMed Res. Int..

[4] Daley C.L., Iaccarino J.M., Lange C., Cambau E., Wallace R.J., Andrejak C., Böttger E.C., Brozek J., Griffith D.E., Guglielmetti L. (2020). Treatment of nontuberculous mycobacterial pulmonary disease: An official ATS/ERS/ESCMID/IDSA clinical practice guideline. Eur. Respir. J..

[5] Haworth C.S., Floto R.A. (2017). Introducing the new BTS Guideline: Management of non-tuberculous mycobacterial pulmonary disease (NTM-PD). Thorax.

[6] Jankovic M., Sabol I., Zmak L., Jankovic V.K., Jakopovic M., Obrovac M., Ticac B., Bulat L.K., Grle S.P., Marekovic I. (2016). Microbiological criteria in non-tuberculous mycobacteria pulmonary disease: A tool for diagnosis and epidemiology. Int. J. Tuberc. Lung Dis..

[7] Prevots D.R., Marras T.K. (2015). Epidemiology of human pulmonary infection with nontuberculous mycobacteria: A review. Clin. Chest Med..

[8] Philley J.V., Griffith D.E. (2015). Treatment of slowly growing mycobacteria. Clin. Chest Med..

[9] Kasperbauer S.H., De Groote M.A. (2015). The treatment of rapidly growing mycobacterial infections. Clin. Chest Med..

[10] Van Ingen J., Aksamit T., Andrejak C., Böttger E.C., Cambau E., Daley C.L., Griffith D.E., Guglielmetti L., Holland S.M., Huitt G.A. (2018). Treatment outcome definitions in nontuberculous mycobacterial pulmonary disease: An NTM-NET consensus statement. Eur. Respir. J..

[11] Wu J., Zhang Y., Li J., Lin S., Wang L., Jiang Y., Pan Q., Shen X. (2014). Increase in Nontuberculous Mycobacteria Isolated in Shanghai, China: Results from a Population-Based Study. PLoS ONE.

[12] Zhang H., Luo M., Zhang K., Yang X., Hu K., Fu Z., Zhang L., Wu P., Wan D., Han M. (2021). Species identification and antimicrobial susceptibility testing of non-tuberculous mycobacteria isolated in Chongqing, Southwest China. Epidemiol Infect..

[13] Zweijpfenning S.M.H., Ingen J.V., Hoefsloot W. (2018). Geographic Distribution of Nontuberculous Mycobacteria Isolated from Clinical Specimens: A Systematic Review. Semin. Respir. Crit. Care Med..

[14] Shen Y., Wang X., Jin J., Wu J., Zhang X., Chen J., Zhang W. (2018). In Vitro Susceptibility of Mycobacterium abscessus and Mycobacterium fortuitum Isolates to 30 Antibiotics. BioMed. Res Int..

[15] Zhou L., Xu D., Liu H., Wan K., Wang R., Yang Z. (2020). Trends in the Prevalence and Antibiotic Resistance of Non-tuberculous Mycobacteria in Mainland China, 2000–2019, Systematic Review and Meta-Analysis. Front. Public Health.

[16] Davies J., Davies D. (2010). Origins and evolution of antibiotic resistance. Microbiol. Mol. Biol. Rev..

[17] Jarand J., Levin A., Zhang L., Huitt G., Mitchell J.D., Daley C.L. (2011). Clinical and microbiologic outcomes in patients receiving treatment for Mycobacterium abscessus pulmonary disease. Clin. Infect. Dis..

[18] Hoefsloot W., van Ingen J., de Lange W.C., Dekhuijzen P.N., Boeree M.J., van Soolingen D. (2009). Clinical relevance of Mycobacterium malmoense isolation in the Netherlands. Eur. Respir. J..

[19] Litvinov V., Makarova M., Galkina K., Khachaturiants E., Krasnova M., Guntupova L., Safonova S. (2018). Drug susceptibility testing of slowly growing non-tuberculous mycobacteria using slomyco test-system. PLoS ONE.

[20] Weng Y.W., Huang C.K., Sy C.L., Wu K.S., Tsai H.C., Lee S.S. (2020). Treatment for Mycobacterium abscessus complex-lung disease. J. Formos. Med. Assoc..

[21] Hatakeyama S., Ohama Y., Okazaki M., Nukui Y., Moriya K. (2017). Antimicrobial susceptibility testing of rapidly growing mycobacteria isolated in Japan. BMC Infect. Dis..

[22] Guo Q., Wei J., Zou W., Li Q., Qian X., Zhu Z. (2021). Antimicrobial susceptibility profiles of Mycobacterium abscessus complex isolates from respiratory specimens in Shanghai, China. J. Glob. Antimicrob Resist..

[23] Hayashi M., Takayanagi N., Kanauchi T., Miyahara Y., Yanagisawa T., Sugita Y. (2012). Prognostic factors of 634 HIV-negative patients with Mycobacterium avium complex lung disease. Am. J. Respir. Crit. Care Med..

[24] Diel R., Lipman M., Hoefsloot W. (2018). High mortality in patients with Mycobacterium avium complex lung disease: A systematic review. BMC Infect. Dis..

[25] Mirsaeidi M., Machado R.F., Garcia J.G., Schraufnagel D.E. (2014). Nontuberculous mycobacterial disease mortality in the United States, 1999–2010, a population-based comparative study. PLoS ONE.

[26] Andréjak C., Thomsen V.Ø., Johansen I.S., Riis A., Benfield T.L., Duhaut P., Sørensen H.T., Lescure F.X., Thomsen R.W. (2010). Nontuberculous pulmonary mycobacteriosis in Denmark: Incidence and prognostic factors. Am. J. Respir. Crit. Care Med..

[27] Fujiwara K., Furuuchi K., Aono A., Uesugi F., Shirai T., Nakamoto K., Shimada T., Mochizuki F., Tanaka Y., Iijima H. (2021). Clinical risk factors related to treatment failure in Mycobacterium abscessus lung disease. Eur. J. Clin. Microbiol. Infect. Dis..

[28] Gochi M., Takayanagi N., Kanauchi T., Ishiguro T., Yanagisawa T., Sugita Y. (2015). Retrospective study of the predictors of mortality and radiographic deterioration in 782 patients with nodular/bronchiectatic Mycobacterium avium complex lung disease. BMJ Open.

[29] Yamakawa H., Takayanagi N., Miyahara Y., Ishiguro T., Kanauchi T., Hoshi T., Yanagisawa T., Sugita Y. (2013). Prognostic factors and radiographic outcomes of nontuberculous mycobacterial lung disease in rheumatoid arthritis. J. Rheumatol..

[30] González-Pérez M., Mariño-Ramírez L., Parra-López C.A., Murcia M.I., Marquina B., Mata-Espinoza D., Rodriguez-Míguez Y., Baay-Guzman G.J., Huerta-Yepez S., Hernandez-Pando R. (2013). Virulence and immune response induced by Mycobacterium avium complex strains in a model of progressive pulmonary tuberculosis and subcutaneous infection in BALB/c mice. Infect. Immun..

[31] Mori S., Koga Y., Nakamura K., Hirooka S., Matsuoka T., Uramoto H., Sakamoto O., Ueki Y. (2020). Mortality in rheumatoid arthritis patients with pulmonary nontuberculous mycobacterial disease: A retrospective cohort study. PLoS ONE.

[32] Kumagai S., Ito A., Hashimoto T., Marumo S., Tokumasu H., Kotani A., Yamaki H., Shirata M., Furuuchi K., Fukui M. (2017). Development and validation of a prognostic scoring model for Mycobacterium avium complex lung disease: An observational cohort study. BMC Infect. Dis..

[33] Hu C., Huang L., Cai M., Wang W., Shi X., Chen W. (2019). Characterization of non-tuberculous mycobacterial pulmonary disease in Nanjing district of China. BMC Infect. Dis..

[34] Eisenberg I., Yasin A., Fuks L., Stein N., Saliba W., Kramer M.R., Adir Y., Shteinberg M. (2020). Radiologic Characteristics of Non-tuberculous Mycobacteria Infection in Patients with Bronchiectasis. Lung.

[35] Gopalaswamy R., Shanmugam S., Mondal R., Subbian S. (2020). Of tuberculosis and non-tuberculous mycobacterial infections-a comparative analysis of epidemiology, diagnosis and treatment. J. Biomed. Sci..

[36] Bai X., Bai A., Honda J.R., Eichstaedt C., Musheyev A., Feng Z., Huitt G., Harbeck R., Kosmider B., Sandhaus R.A. (2019). Alpha-1-Antitrypsin Enhances Primary Human Macrophage Immunity Against Non-tuberculous Mycobacteria. Front. Immunol..

[37] López C.M., Gallego C.L., Calvo J.C., Vasallo I.J.T., Ramírez M.T.R. (2021). Patients with non-tuberculous mycobacteria in respiratory samples: A 5-year epidemiological study. Rev. Esp. Quimioter..

